# Borderline personality disorder and Mental Health Services: from the diagnostic pathway to the implementation of good practices. A real-world feasibility study

**DOI:** 10.1192/j.eurpsy.2026.10999

**Published:** 2026-07-31

**Authors:** A. Zucchetti, A. Cassandra Julia, B. Deborah, G. Agnese, B. Stefano, V. Antonio

**Affiliations:** 1 Clinical and Experimental Sciences, University of Brescia; 2 Mental Health and Addiction Services, ASST Spedali Civili, Brescia, Italy

## Abstract

**Introduction:**

Borderline Personality Disorder (BPD) is a mental disorder that impacts psychosocial functioning, typically resulting in reduced occupational and educational achievement, inability to maintain long-term relationships, increased conflict between partners, and low levels of social support. Patients and family members also report low satisfaction in quality of life. Treatment requires a multidisciplinary approach: psychopharmacological therapy, psychotherapy, psychosocial therapies and caregivers support.

**Objectives:**

The aim of this study is to explore the feasibility of good practice care in terms of changes in symptom severity and psychosocial functioning in a sample of patients with BPD in a real-world clinical setting.

The study also examines whether training of family members has an impact on the perceived burden of caring and on the development of skills for managing their relative’s disorder.

**Methods:**

Fifteen BPD patients at first contact with a Mental Health Service (MHS) of the Department of Mental Health of Spedali Civili Hospital, Brescia were recruited and underwent the following assessment: Toronto Alexithymia Scale (TAS-20), Barratt Impulsiveness Scale (BIS-11), Difficulties in Emotion Regulation Scale (DERS), Zanarini Rating Scale for BPD (ZAN-BPD), Personal and Social Performance Scale (PSP), and Health of the Nation Outcome Scale (HONOS), before and after the interventions. All the patients participated for 9 months in the following evidence-based (EB) interventions for BPD: DBT skills training and psychoeducation with Good Psychiatric Management. A group of patients’ relatives (N=6) underwent for 3 months the Family Connection (FC) group.

Statistical data were calculated using Rstudio software.

**Results:**

Patients (N=14, one drop out) demonstrated improvement in clinical dimensions (TAS-20 p<0.05; BIS-11 p<0.05; DERS p<0.05; ZAN-BPD p<0.001) and in psychosocial functioning (PSP p<0.05; HONOS p<0.05) after the integrated treatment (Wilcoxon test). Patients were then divided into two groups, based on whether or not their parents had completed the FC programme, no significant changes between the two groups were found. A decrease in the perceived burden on family members was observed, although this result did not reach statistical significance.

**Image 1:**
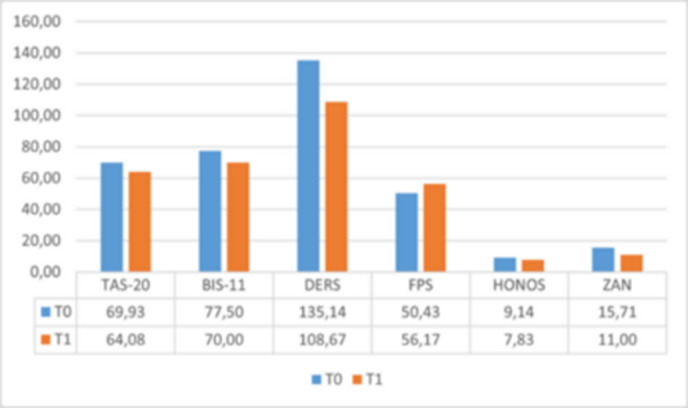
Image 1: Long description.

**Conclusions:**

Findings highlight that EB interventions for BPD are feasible and sustainable in a clinical real-world setting. Although preliminary, results indicate a clinical and a psychosocial functioning improvement in individuals with BPD. Starting from these explorative data we argue that EB interventions should be implemented in MHS. The hypothesis is also that involving the family not only helps family members themselves, but also patients. Currently, the small sample size lacks statistical power, but we’ll expect that these results will be confirmed in a larger sample.

**Disclosure of Interest:**

None Declared

## Long descriptions

### Image 1: Long description

A clustered bar chart with a data table below it. The y-axis represents numerical scores ranging from 0.00 to 160.00 in increments of 20.00. The x-axis lists six categories: T A S-20, B I S-11, D E R S, F P S, H O N O S, and Z A N. Each category has two bars: a blue bar for T 0 and an orange bar for T 1.

Data values from the integrated table:

* T A S-20: T 0 is 69.93 and T 1 is 64.08, showing a slight decrease.

* B I S-11: T 0 is 77.50 and T 1 is 70.00, showing a decrease.

* D E R S: T 0 is 135.14 and T 1 is 108.67, showing the most significant decrease.

* F P S: T 0 is 50.43 and T 1 is 56.17, showing a slight increase.

* H O N O S: T 0 is 9.14 and T 1 is 7.83, showing a slight decrease.

* Z A N: T 0 is 15.71 and T 1 is 11.00, showing a decrease.

The legend at the bottom indicates blue represents T 0 and orange represents T 1.

Navigate back to Image 1:.

